# Aurora kinase A stabilizes FOXM1 to enhance paclitaxel resistance in triple‐negative breast cancer

**DOI:** 10.1111/jcmm.14538

**Published:** 2019-07-30

**Authors:** Na Yang, Chang Wang, Jian Wang, Zifeng Wang, Di Huang, Min Yan, Muhammad Kamran, Quentin Liu, BangLao Xu

**Affiliations:** ^1^ Department of Laboratory Medicine, Guangzhou First People's Hospital, School of Medicine South China University of Technology Guangzhou China; ^2^ Department of Pathology, GanZhou Municipal People's Hospital NanChang University GanZhou China; ^3^ State Key Laboratory of Oncology in South China, Cancer Center Sun Yat‐sen University Guangzhou China; ^4^ Department of Breast Surgery, Guangzhou First People's Hospital, School of Medicine South China University of Technology Guangzhou China

**Keywords:** Aurora kinase A, cell cycle, chemotherapy, FOXM1, paclitaxel resistance, triple‐negative breast cancer

## Abstract

Triple‐negative breast cancer (TNBC) has a relatively poor outcome. Acquired chemoresistance is a major clinical challenge for TNBC patients. Previously, we reported that kinase‐dead Aurora kinase A (Aurora‐A) could effectively transactivate the FOXM1 promoter. Here, we demonstrate an additional pathway through which Aurora‐A stabilizes FOXM1 by attenuating its ubiquitin in TNBC. Specifically, Aurora‐A stabilizes FOXM1 in late M phase and early G1 phase of the cell cycle, which promotes proliferation of TNBC cells. Knock‐down of Aurora‐A significantly suppresses cell proliferation in TNBC cell lines and can be rescued by FOXM1 overexpression. We observe that paclitaxel‐resistant TNBC cells exhibit high expression of Aurora‐A and FOXM1. Overexpression of Aurora‐A offers TNBC cells an additional growth advantage and protection against paclitaxel. Moreover, Aurora‐A and FOXM1 could be simultaneously targeted by thiostrepton. Combination of thiostrepton and paclitaxel treatment reverses paclitaxel resistance and significantly inhibits cell proliferation. In conclusion, our study reveals additional mechanism through which Aurora‐A regulates FOXM1 and provides a new therapeutic strategy to treat paclitaxel‐resistant triple‐negative breast cancer.

## 1 INTRODUCTION

Triple‐negative breast cancer (TNBC) represents 15%–20% of newly diagnosed breast carcinoma and is characterized by low expression of progesterone receptor (PR), oestrogen receptor (ER) and human epidermal growth factor receptor 2 (HER2).^1^, ^2^, ^3^ Patients with TNBC have a relatively poor outcome and cannot be treated with endocrine therapy or therapies targeted to HER2. Thus, to date, chemotherapy remains the only possible therapeutic option in the treatment of TNBC.^4^, ^5^ Paclitaxel is one of the most frequently used chemotherapeutic drug for TNBC.^6^ It disrupts normal microtubule dynamics and arrests cancer cells in mitosis. Although TNBC patients are initially responsive to paclitaxel, acquired chemoresistance is inevitable and put forward a major clinical challenge for patients that relapsed.^7^, ^8^ Thus, to elucidate the molecular mechanism of paclitaxel resistance and develop new combination drug therapy for TNBC are urgently needed to decrease TNBC‐related mortality.

Aurora kinase A (Aurora‐A) is a member of a serine/threonine kinase family involved in mitotic cell division and genetic instability.^9^ Overexpression of Aurora‐A has been reported to participate in multi‐drug resistance.^10^, ^11^, ^12^ Several studies have shown that aberrant expression of Aurora‐A is highly correlated with triple‐negative breast cancer (TNBC) and was a potential therapeutic target for TNBC.^3^, ^13^, ^14^, ^15^ Aurora‐A exerts its function in cancer through kinase‐dependent and kinase‐independent manner. In human neuroblastoma, Aurora‐A stabilizes N‐Myc and contributes to the development of neuroblastoma independently of its kinase activity.^16^ Aurora‐A was also reported to phosphorylate YAP and promotes YAP‐mediated transcription in TNBC.^15^ We reported previously that kinase‐dead Aurora‐A can play as a transcriptional co‐factor in nucleus to transactivate the FOXM1 promoter in breast cancer stem cell.^17^ FOXM1 was reported to mediate drug resistance and was highly up‐regulated in TNBC.^18^, ^19^ To elucidate the underlying mechanism for Aurora‐A regulating FOXM1 in TNBC is important for the targeted therapy that aiming to block the critical oncogenic function of Aurora‐A.

In the present study, we demonstrate that Aurora‐A binds and stabilizes FOXM1 protein independently of its kinase activity. This function is critical for the growth of TNBC cells. We observed that overexpression of Aurora‐A mediated paclitaxel resistance in TNBC cells. Moreover, Aurora‐A and FOXM1 could be simultaneously targeted by thiostrepton which could overcome paclitaxel resistance in TNBC cells. Finally, aberrant expression of FOXM1 and Aurora‐A was found highly correlated in human triple‐negative breast cancer sample. Overall, our studies revealed additional mechanism through which Aurora‐A regulates FOXM1 and provided a new combination drug therapeutic strategy to treat paclitaxel‐resistant triple‐negative breast cancer.

## 2 MATERIALS AND METHODS

### 2.1 Cell culture and cell cycle arrest

Human breast cancer cell lines MDA‐MB‐231 and MCF‐7 were acquired from the American Type Culture Collection (Beijing Zhongyuan Ltd) and cultured in media as recommended by the provider. Paclitaxel‐resistant MDA‐MB‐231 cells were cultured in Dulbecco's modified Eagle's medium (Gibco, ThermoFisher Scientific) supplemented with 10% foetal bovine serum (Hyclone, ThermoFisher Scientific). The paclitaxel‐resistant cell line is derived from parental MDA‐MB‐231 cells. MDA‐MB‐231 cells were subjected to increasing concentrations of paclitaxel (T1912, Sigma) until the cells acquire resistance to 0.2 mol/L of paclitaxel. The paclitaxel resistance cell was then maintained in 0.2 mol/L of paclitaxel. For double thymidine block, cells were arrested with 2 mmol/L thymidine (Sigma) medium for 19 hours. After cells were washed three times with phosphate‐buffered saline (PBS) and released into fresh medium for 9 hours, they were then treated with thymidine for an additional 16 hours. To synchronize cells in G2/M phase, cells were treated with 40 ng/mL nocodazole (Sigma) for 16 hours.

### 2.2 In vivo ubiquitination assays and colony formation assay

Cells were transfected with HA‐ubiquitin and pcDNA3‐FOXM1, with either Aur‐A or empty vector. After that, cells were treated with MG132 for the last 6 hours of transfection and lysed with ubiquitin assay buffer (6 mol/L guanidine‐HCl, 0.1 mol/L Na2HPO4/Na2HPO4, 0.01 mol/L Tris‐HCl, 5 μmol/L imidazole, 10 μmol/L β‐mercaptoethanol). Ubiquitinated proteins were eluted with elution buffer (200 μmol/L imidazole, 0.15 mol/L Tris‐HCl, pH 6.7, 30% glycerol, 0.72 M‐mercaptoethanol, 5% sodium dodecyl sulphate). The presence of ubiquitinated FOXM1 was analysed by immunoblotting with FOXM1 antibody.^20^ For colony‐forming assay, 3 to 5 × 10^3^ cells were plated in triplicate in 6 cm plate. Twenty‐four hours later, treatment was initiated. After 14 to 17 days, cells were fixed and stained with crystal violet. Quantification was done using Adobe Photoshop. All *P* values were calculated using the Student's *t* test.

### 2.3 Gene knock‐down using shRNA and siRNA

Gene silencing was performed using specific shRNAs delivered by a lentiviral system acquired from Sigma‐Aldrich (Shanghai, China), following the instructions provided. Briefly, to yield lentiviruses containing specific shRNA sequences, 293T cells were cotransfected with 2.5 μg pMD2.G and 7.5 μg psPAX2 packaging plasmids and 10 μg of the pLKO.1 plasmid containing the specific shRNA for 24 hours. The lentivirus containing cultured medium was collected and stored at −80℃ as aliquots until further use. To deliver the specific shRNA construct, approximately 10% confluent cells were incubated with the lentivirus bearing specific shRNA in growth medium containing 8 mg/mL polybrene at 37℃ for 24 hours. The transduced cells were then selected with 2 mg/mL puromycin. For small interfering RNA assay, cells were cultured for 16 hours in 6‐well plates before transfection. The siRNA oligonucleotides were as follows:

si‐Aur‐A‐1:5′‐AUGCCCUGUCUUACUGUCA‐3′;

si‐Aur‐A‐2:5′‐GGCAACCAGTGTACCTCAT‐3′;

si‐Aur‐A‐3:5′‐ATTCTTCCCAGCGCGTTCC‐3′;

si‐FOXM1‐1:5′‐GCACTATCAACAATAGCCTAT‐3′;

si‐FOXM1‐2:5′‐GCCAATCGTTCTCTGACAGAA‐3′;

si‐FOXM1‐3:5′‐GGACCACUUUCCCUACUUU‐3′;

control: 5′‐UUCUCCGAACGUGUCACGU‐3′.

siRNA was transfected into cells using Lipofectamine 2000 (Invitrogen) according to the manufacturer's instructions. To confirm down‐regulation efficacy, targeted genes were detected by Western blot.

### 2.4 IP and Western blot analysis

Cells were harvested and lysed in immunoprecipitation (IP) buffer on ice for 20 minutes. The IP buffer consisted of 50 mmol/L Tris (pH 7.5), 100 mmol/L NaCl, 5 mmol/L EDTA, 5 mmol/L EGTA, 1% NP‐40, 5% glycerol, freshly added protease inhibitor cocktail, 2 mmol/L phenylmethylsulfonyl fluoride, and 2 mmol/L NaF and 2 mmol/L NaVO4 as phosphatase inhibitors. Cell extracts were clarified by centrifugation at 12 000 *g* for 5 minutes at 4°C. Protein concentrations were determined by the Bradford method with the Bio‐Rad protein assay reagent. For Western blot, cells were lysed in RIPA buffer and the protein concentrations determined by the Bradford assay. Equal amounts of cell extracts were subjects to electrophoresis on sodium dodecyl sulphate‐polyacrylamide gel and blotted onto nitrocellulose membrane (Millipore). After protein transfer, the membranes were blocked and then incubated with glyceraldehyde 3‐phosphate dehydrogenase (Ambion, ThermoFisher Scientific). The antibodies used were as follows: p‐AURKA (Thr288; Cell Signaling Technology, Gene Company Ltd), AURKA (Upstate, Gene Company Ltd), HA‐tag (Sigma), FOXM1 (Santa Cruz, C‐20) and GAPDH (Epitomics, Abcam). Membrane was incubated in primary antibodies at 4°C overnight. The membranes were then incubated for 1 hour at room temperature with the appropriate secondary antibodies. Proteins were detected with an enhanced chemiluminescence kit (Pierce, ThermoFisher Scientific).

### 2.5 Real‐time RT–PCR

Total RNA was extracted using TRIzol reagent (Invitrogen), and used for generating cDNA using SuperScript III RT (Invitrogen) in the presence of oligo‐dT primers. Real‐time reverse transcriptase‐PCR was performed using Platinum SYBR Green qPCR Super‐Mix (Invitrogen), with GAPDH (glyceraldehyde 3‐phosphate dehydrogenase) as the internal control.

### 2.6 Immunohistochemical staining

Following informed consent and in accordance with the guidelines of the Institutional Review Boards, breast cancer specimens were collected from patients undergoing surgery at the Guangzhou first people's hospital, China. Paraffin‐embedded tissue blocks were sectioned for immunohistochemistry as described previously. The deparaffinized sections were incubated in H_2_O_2_ (3%) for 10 minutes, blocked in 1% bovine serum albumin for 60 minutes and incubated with an anti‐AURKA antibody (1:200 dilution) or an anti‐FOXM1 antibody (1:100 dilution) at 4°C overnight. ImageJ software was used to measure FOXM1 and Aurora‐A scoring. The degree of score (0 to 3+) is based on both image intensity and completeness of staining in tumour cells. The 1+ score was considered as low expression, and the score ≥ 2+ was considered as high expression.

### 2.7 Statistical analysis

Statistical analyses were performed using the SPSS software, version 16.0 (SPSS Inc) and with GraphPad Prism 5.0 (GraphPad Software Inc). The unpaired Student's *t* test was used to perform statistical analysis between two groups. Continuous and categorical variables were compared between groups, using the Mann‐Whitney test and one‐way ANOVA for non‐parametric continuous data. Pearson's correlation coefficient (*r*) was used to describe the correlation between two variables. A value of *P* < .05 was considered statistically significant. The UALCAN web resource was used for analysing cancer transcriptome data in TCGA database.^21^

## 3 RESULTS

### 3.1 Aurora‐A and FOXM1 are overexpressed and correlated in triple‐negative breast cancer

**Figure 1:**
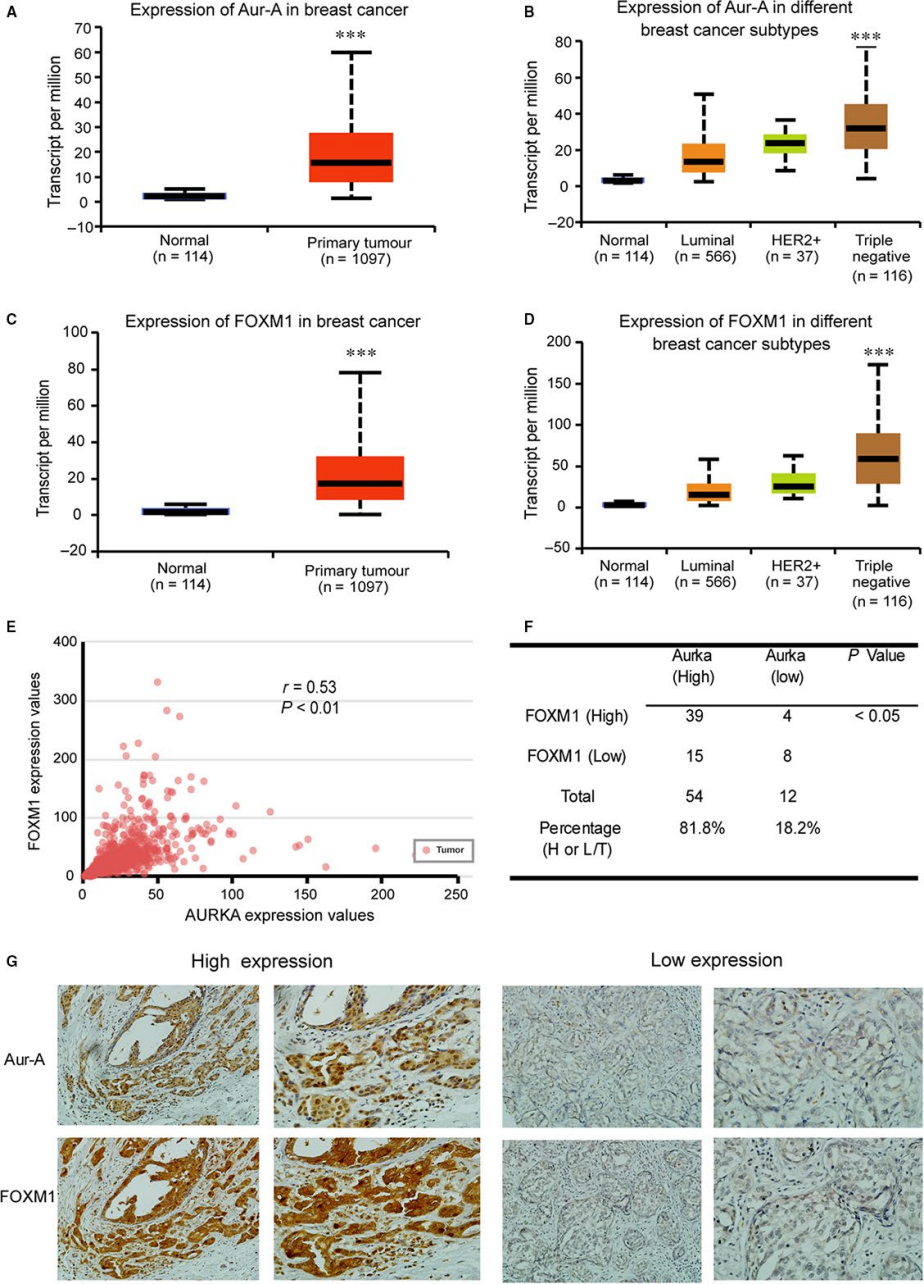
Aurora‐A and FOXM1 are overexpressed and correlated in triple‐negative breast cancer. A, Transcript of Aurora‐A was evaluated in breast cancer in TCGA database. Triple‐negative breast cancer is defined by low or no expression of progesterone receptor (PR), oestrogen receptor (ER) and human epidermal growth factor receptor 2 (HER2). B, Transcript of Aurora‐A was evaluated in different breast cancer subtypes in TCGA database. C, Transcript of FOXM1 was evaluated in breast cancer in TCGA database. D, Transcript of FOXM1 was evaluated in different breast cancer subtypes in TCGA database. E, The correlation between Aurora‐A and FOXM1 in TNBC was evaluated in TCGA database by Pearson's correlation test. F, FOXM1 and Aurora‐A levels were evaluated in 66 TNBC specimens. Statistical comparisons were made using chi‐square test. G, Expression of Aurora‐A and FOXM1 was examined via IHC staining in TNBC specimens. Representative picture was shown

To investigate the significance of Aurora‐A and FOXM1 in breast cancer, we evaluate cancer transcriptome in TCGA database at UALCAN web resource.^21^ As shown in Figure 1A, Aurora‐A is overexpressed in clinical breast cancer sample, with significantly higher in triple‐negative breast cancer (Figure 1B). Consistently, FOXM1 is also overexpressed in clinical breast cancer sample and significantly higher in triple‐negative breast cancer (Figure 1C,D). There is a significant positive correlation between Aurora‐A and FOXM1 in breast cancer (*r* = .53, *P* < .01; Figure 1E). Next, we analysed the expression level of Aurora‐A and FOXM1 in a panel of 66 TNBC samples and 11 benign breast tumour samples from GuangZhou First People’ hospital. Aurora‐A was highly expressed in 54 samples and lowly expressed in 12 samples while FOXM1 was highly expressed in 43 samples and lowly expressed in 23 samples. Aurora‐A and FOXM1 were co‐expressed in 39 TNBC samples, which was consistent with the TCGA database (*P* < .05; Figure 1F). The representative picture of high expression and low expression was shown in Figure 1G. Together, our data suggest that Aurora‐A and FOXM1 are co‐expressed in clinical TNBC samples, supporting the critical role of Aurora‐A and FOXM1 in TNBC growth.

### 3.2 Aurora‐A stabilizes FOXM1 during cell cycle

**Figure 2:**
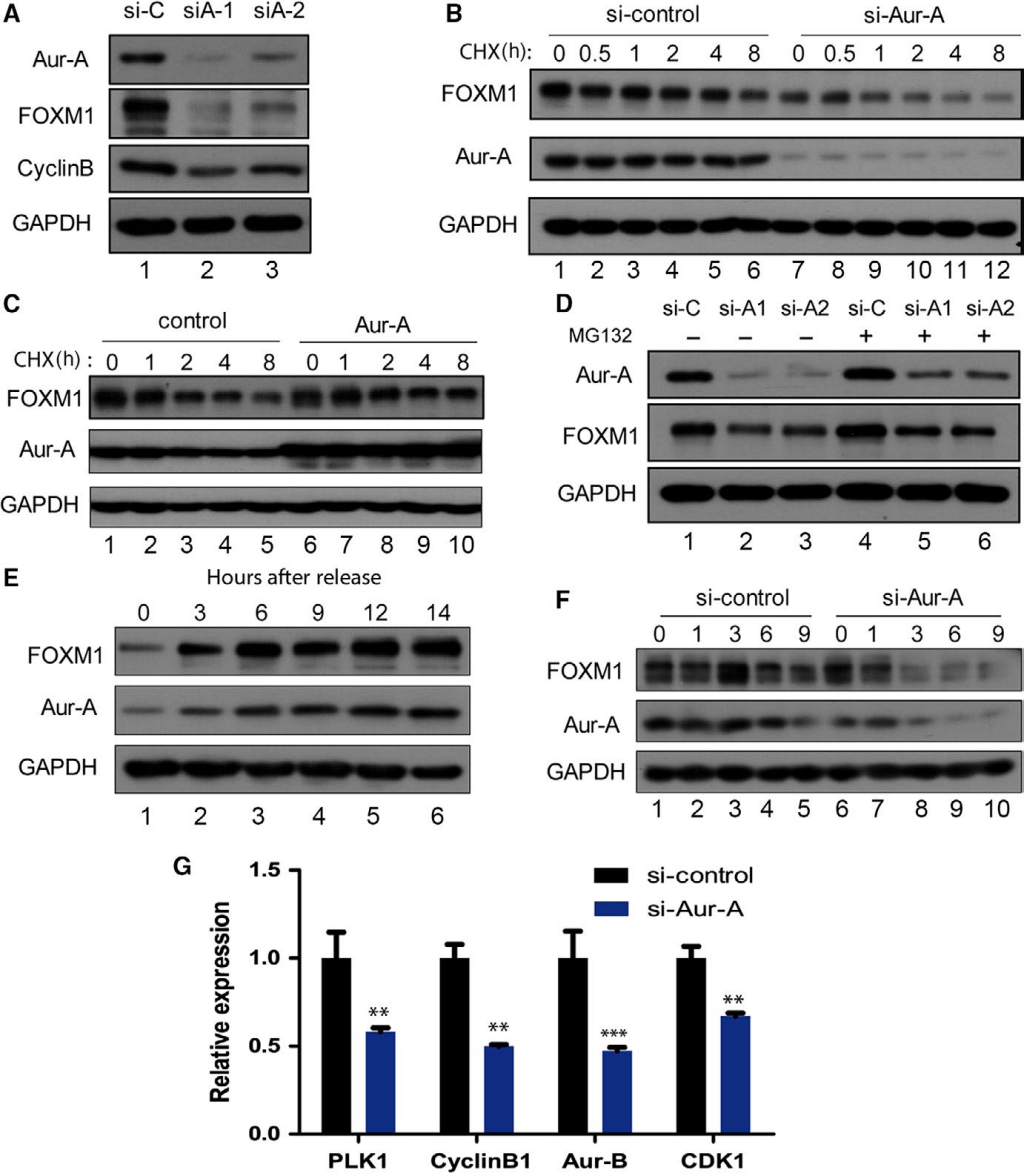
Aurora‐A stabilizes FOXM1 protein during cell cycle in late M phase and early G1 phase. A, Western blot analysis with indicated antibodies in control (si‐C) and Aurora‐A knock‐down (si‐A) MDA‐MB‐231 cells. B, Western blots analysis of the FOXM1 stability in MDA‐MB‐231 cells expressing either control siRNA or siRNA targeting Aurora‐A. Cells were treated with cycloheximide (CHX). Lysates were prepared at the indicated times after addition of CHX and probed with the indicated antibodies. C, Stabilization of FOXM1 by expression of Aurora‐A. Cells were transfected with control vectors and Aurora‐A overexpression vectors as indicated. D, Cells were transfected with si‐control and si‐Aurora‐A small RNA. Subsequently, MG‐132 was added to cells at last 6 h and lysates were analysed by Western blot. E, Cells were arrested at the G1‐S boundary by double thymidine block, released into fresh medium, and harvested at the indicated times. Cell lysates were subjected to Western blot assays with indicated antibodies. F, Cells were released from nocodazole block (prometaphase) for the indicated times. Cell extracts were subjected to Western blotting with indicated antibody. G, Cells were transfected with si‐control and si‐Aurora‐A small RNA, and real‐time RT‐PCR was performed to evaluate the M phase cell cycle genes

Next, we test whether Aurora‐A regulates FOXM1 in TNBC cells, MDA‐MB‐231 cells were transfected with Aurora‐A siRNA or a control siRNA. Immunoblots showed that depletion of Aurora‐A obviously led to a decrease of FOXM1 protein levels, which could account for the reduced expression of FOXM1 target gene cyclinB1 (Figure 2A, lane 2 and lane 3). We speculated that Aurora‐A regulates the protein stability of FOXM1. Control and si‐Aurora‐A cells were treated with cycloheximide to block new protein synthesis and were harvested at different time‐points afterwards. Under these conditions, depletion of Aurora‐A obviously reduced the half‐life of endogenous FOXM1 (Figure 2B, lane 4, 5, 6 vs lane 10, 11, 12). Conversely, transient transfection of Aur‐A expression vectors in 231 cells enhanced steady‐state levels of FOXM1, which indicated an increase in FOXM1 stability (Figure 2C, lane 3, 4, 5 vs lane 8, 9, 10). To further test Aurora‐A‐mediated FOXM1 stability, MG132 was incubated in medium to prevent degradation by the 26S proteasome. The protein level of FOXM1 was partly rescued by adding MG132 (Figure 2D, lane 2, 3 vs lane 5, 6). Because Aurora‐A and FOXM1 were critical cell cycle protein of G2/M phase, we test whether Aurora‐A regulates FOXM1 during cell cycle. After cells were synchronized in G1/S phase by a double thymidine block, protein extracts were prepared at indicated time‐point following thymidine removal and analysed by immunoblotting. As shown in Figure 2E (lane 2 to 6), the level of FOXM1 increased during the late S phase (after 3 hours of thymidine removal) and the transition between G2 phase and mitosis (at the time‐point of 9‐12 hours). Aurora‐A and FOXM1 were simultaneously accumulated gradually from S phase to G2 phase and peaked in mitosis. Interestingly, both Aurora‐A and FOXM1 were not degraded at the late M phase and early G1 phase (at the time‐point of 14 hours). This result suggested that Aurora‐A and FOXM1 undergone almost identical changes during cell cycle and they were stable at late M phase and early G1 phase in TNBC cells, which was different from other cells.^20^ To further investigate the role of Aurora‐A in the stabilization of the FOXM1 protein during cell cycle, we performed siRNA transfection experiments with synchronized MDA‐MB‐231 cells. Cells were synchronized in M phase by treating them with nocodazole for 16 hours (Figure 2F). After nocodazole was removed, the cells were allowed progression through the G1 and S phases. The synchronized cells at indicated time‐point were harvested. Depletion of Aurora‐A in MDA‐MB‐231 cells obviously reduced the levels of FOXM1 protein after 3 hours of nocodazole removal (a time‐point corresponding to late M phase), suggesting a role of Aurora‐A in the stabilization of FOXM1 in the late M and early G1 phases of the cell cycle. Furthermore, down‐regulation of Aurora‐A by siRNA significantly reduced the downstream genes of FOXM1 in M phase (Figure 2G). Taken together, the regulation of Aurora‐A on FOXM1 in the cell cycle of TNBC cells is not restricted to the G2/M phase when the kinase activity of Aurora‐A is highest, but also in the M/G1 transition.

### 3.3 Aurora‐A up‐regulates FOXM1 in triple‐negative breast cancer via kinase‐independent manner

**Figure 3:**
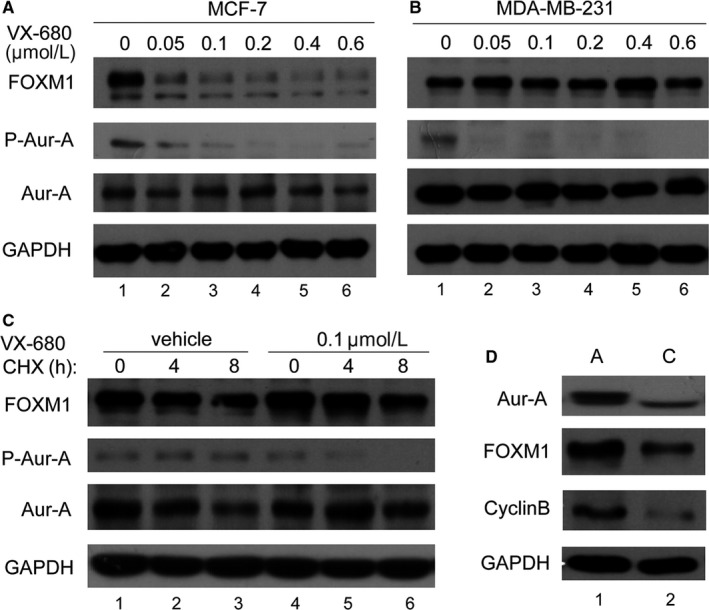
Aurora‐A up‐regulates FOXM1 in triple‐negative breast cancer in kinase‐independent manner. A, MCF‐7 cells were treated with indicated dose of VX680 and subjected to Western blot analysis to determine the protein expression levels. B, MDA‐MB‐231 cells were treated with indicated dose of VX680, and Western blot analysis was done to determine the protein expression levels. C, MDA‐MB‐231 cells were treated with VX680 (0.6 μmol/L) for 8 h, and after that, cells were co‐treated with cycloheximide (CHX) at the indicated time. Lysates were prepared and probed with the indicated antibodies. D, MDA‐MB‐231 cells were transfected with the indicated control vectors and Aur‐A expression vectors. Western blot analysis was done to determine the protein expression levels

Previously, we reported that Aurora‐A regulated FOXM1 at transcription level via kinase‐independent manner was essential for breast cancer stem cell.^17^ To test whether the kinase activity of Aurora‐A is essential for the degradation of the FOXM1 protein in TNBC, we inhibited the kinase activity of Aurora‐A by treating MDA‐MB‐231 and MCF‐7 cells with Aurora‐A inhibitor VX680. Treatment of VX680 caused obviously decreased FOXM1 level in MCF‐7 cells, whereas in MDA‐MB‐231 cells there is no obvious reduction in FOXM1 levels at indicated concentration (Figure 3A lane 2‐6 vs 3B lane 2‐6). Since MCF‐7 is ER‐positive cells, we suggest that kinase activity is important for Aurora‐A regulating FOXM1 in ER‐positive cells, whereas in TNBC cells it is kinase independent. To further investigate the requirement of Aurora‐A kinase activity in the degradation of the FOXM1 protein, cells were simultaneously treated with cycloheximide and VX680 or cycloheximide alone to block new protein synthesis and kinase activity. The result showed there was no significant difference in FOXM1 level between the VX680 treated cells and control cells (Figure 3C, lane 2, 3 vs lane 5, 6). Consistently, overexpression of Aurora‐A up‐regulated FOXM1 and its downstream factor cyclinB1 in MDA‐MB‐231 cells (Figure 3D, lane 2).

### 3.4 Aurora‐A directly binds and attenuates ubiquitin of FOXM1

**Figure 4:**
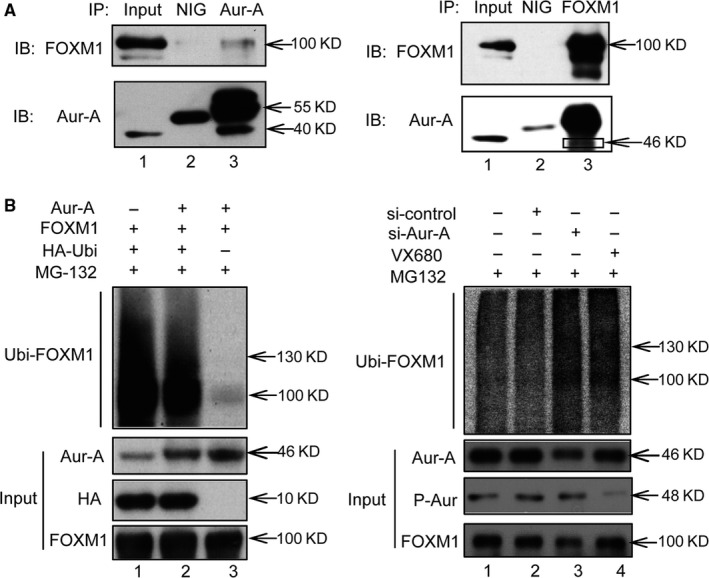
Aurora‐A directly binds and attenuates ubiquitin of FOXM1. A, IP assay was performed to detect the interaction of endogenous Aurora‐A and FOXM1 in MDA‐MB‐231. B, Cells were transfected with HA‐ubiquitin (HA‐ubi) and FOXM1 expression vector along with Aurora‐A expression vector or empty vector. MG‐132 (25 mmol/L) was added to block proteasomal degradation. Lysates of the cells were subjected to IP using HA antibody. The ubiquitinated proteins were analysed for polyubiquitination of the FOXM1 protein by Western blot assays. C, Cells were transfected with siRNA against Aurora‐A or with control siRNA or treated with VX680. Four hours before harvesting, MG‐132 (25 mmol/L) was added to block proteasomal degradation. Lysates of the cells were subjected to IP using FOXM1 antibody. Western blot was performed to analyse the ubiquitin status of FOXM1

To explore the mechanisms by which knock‐down of Aurora‐A reduced FOXM1 protein, we conducted IP assays to test whether Aurora‐A interacted with FOXM1 protein. IP assays showed that there is a specific interaction between Aurora‐A and the FOXM1 protein (Figure 4A, lane 3), supporting the notion that FOXM1 is a target of Aurora‐A. Next, we performed in vivo ubiquitination assays to see whether Aurora‐A regulates the ubiquitin of FOXM1. 293T cells were transfected with HA‐ubiquitin and FOXM1 expression vectors along with either empty vector or Aurora‐A expression vector. Twenty‐four hours following transfection, cells were treated with MG132 for 6 hours. The levels of ubiquitinated FOXM1 protein were analysed by Western blot analysis using FOXM1 antibody. As shown in Figure 4B (lane 1 vs lane 2), expression of Aurora‐A decreased the ubiquitination of FOXM1. To analyse the endogenous proteins, cells were depleted of Aurora‐A by siRNA transfection. The cells were treated with MG132 for 6 hours before being harvested and subjected to IP assays using either antibody against FOXM1 or control mouse immunoglobulin G, and immunoprecipitates were analysed by Western blotting with ubiquitin antibody. Clearly, both depletion of Aurora‐A and inhibition of its kinase activity caused increased ubiquitination of FOXM1 (Figure 4C, lane 3 and lane 4). Interestingly, inhibition of Aurora‐A kinase activity also increased the ubiquitination of FOXM1, indicating that the kinase activity of Aurora‐A may decrease the ubiquitination of FOXM1 in triple‐negative breast cancer.

### 3.5 Aurora‐A and FOXM1 are essential for the growth of triple‐negative breast cancer

**Figure 5:**
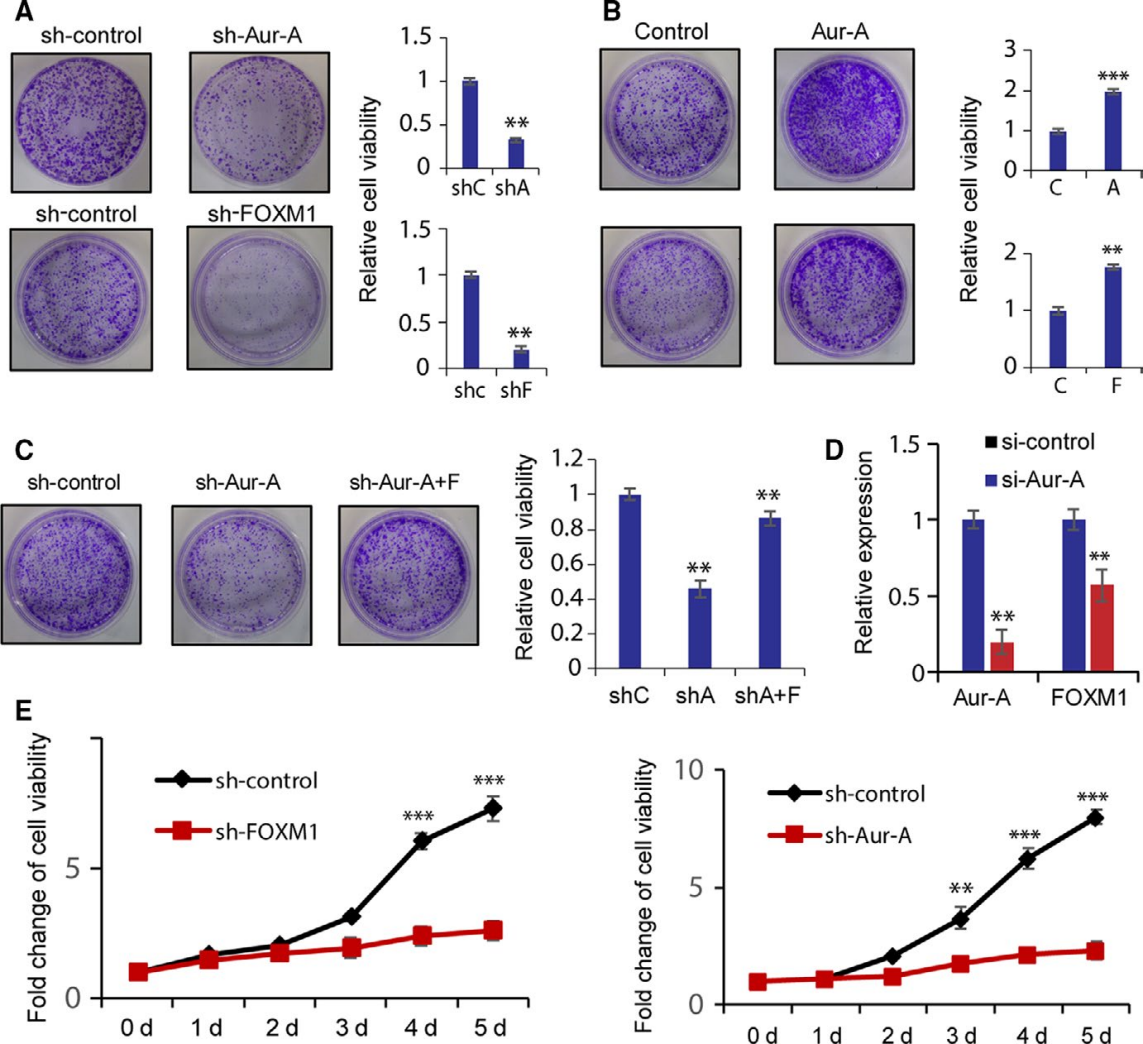
Aurora‐A and FOXM1 are essential for the growth of triple‐negative breast cancer. A, The effect of depletion of Aurora‐A and FOXM1 on MDA‐MB‐231 cells was tested by colony assays using shRNA. B, The effect of overexpression of Aurora‐A and FOXM1 on MDA‐MB‐231 cells was tested by colony assays using expression vectors. C, The sh‐Aurora‐A cell line was transfected with empty or FOXM1 overexpression vectors. Colony formation assay was used to test the cell proliferation. Three plates were counted and averaged. Error bars show standard deviation of triplicate samples. D, After RNA interfering by control siRNA and Aurora‐A‐specific siRNA, the mRNA levels of Aurora‐A and FOXM1 were analysed by real‐time quantitative PCR normalized with GAPDH mRNA levels. E, Growth curve of MDA‐MB‐231 cells infected with control shRNAs or shRNAs targeting Aur‐A or FOXM1. Cells were counted at indicated time to evaluate the cell viability. Columns, mean of three independent experiments in triplicate; bars, ±SD. **P < .01, ***P < .001. Representative wells were shown

To investigate whether the growth of TNBC cells was dependent on Aurora‐A and FOXM1, we designed retroviral shRNA vectors targeting Aurora‐A and FOXM1 and tested them in MDA‐MB‐231 cells. After transfection of sh‐Aurora‐A and sh‐FOXM1 vector, the number of colonies was significantly reduced compared with control cells (Figure 5A). Conversely, elevation of Aurora‐A and FOXM1 levels using recombinant retroviruses significantly increased the number of colony formation (Figure 5B). Next, we investigate whether FOXM1 is essential for Aurora‐A‐mediated inhibition of growth in TNBC cells. MDA‐MB‐231 cells were simultaneously transfected with sh‐Aurora‐A and FOXM1 overexpression vector. The result showed that colony formation was rescued by FOXM1 overexpression, indicating that the reduction in FOXM1 levels is the critical mechanism by which depletion of Aurora‐A inhibits proliferation (Figure 5C). To provide additional evidence that Aurora‐A and FOXM1 is necessary for growth and survival of TNBC cells, MAD‐MB‐231 cells transfected with sh‐Aurora‐A and sh‐FOXM1 were seeded on 12‐well plate, and after that, cells were harvested for cell counting at indicated time‐point. The growth curves showed that both expression of sh‐Aurora‐A and sh‐FOXM1 significantly inhibited proliferation of MDA‐MB‐231 cells (Figure 5E). Furthermore, we found that depletion of Aurora‐A in MDA‐MB‐231 cells reduced the mRNA levels of FOXM1 (Figure 5D), arguing that Aurora‐A regulates FOXM1 levels via both transcription and posttranscriptional mechanisms in TNBC cells. We concluded that Aurora‐A up‐regulating FOXM1 was essential for the growth of TNBC cells.

### 3.6 Aurora‐A enhances paclitaxel resistance in triple‐negative breast cancer

**Figure 6:**
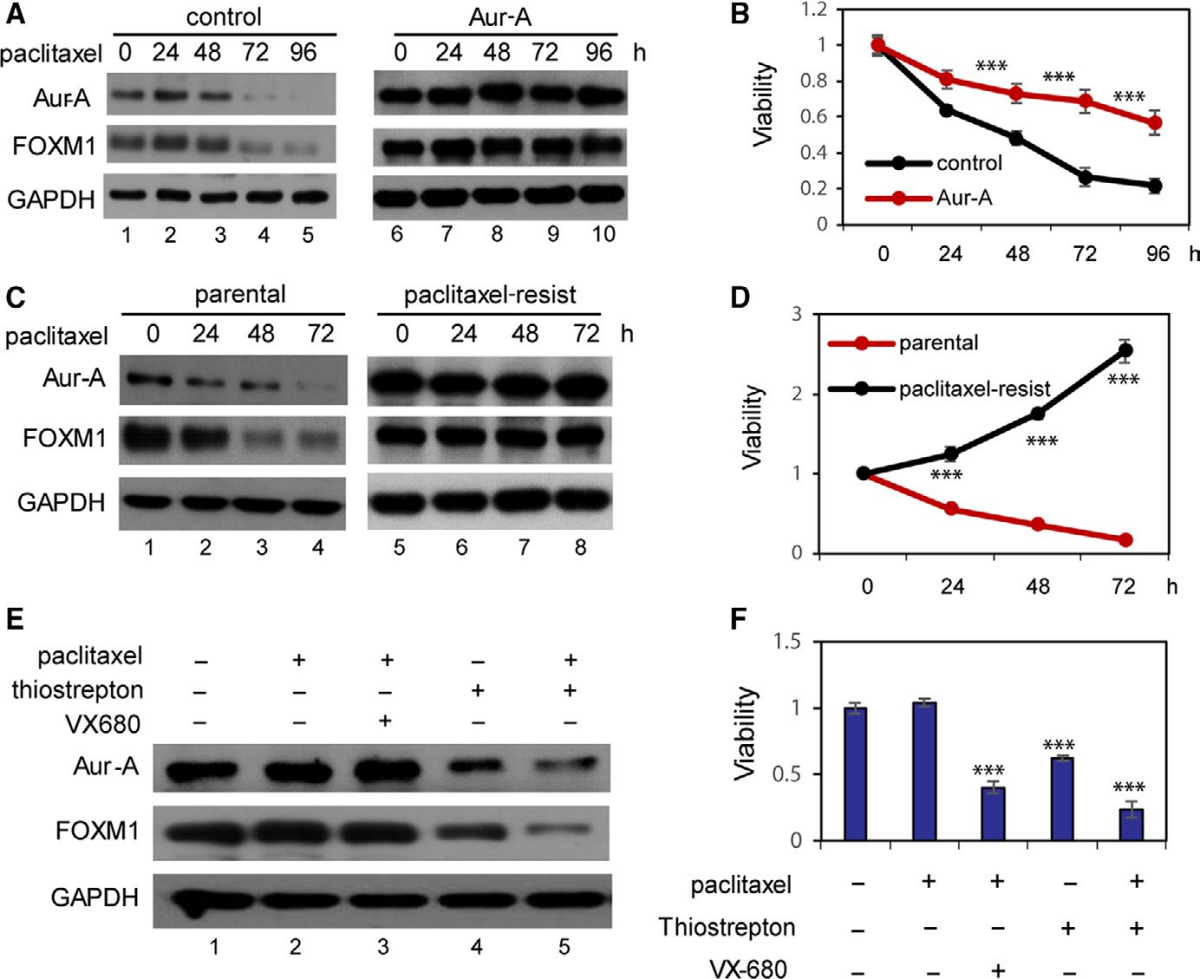
Aurora‐A enhances paclitaxel resistance in triple‐negative breast cancer. A, Control cells and Aurora‐A overexpression cells were treated with 0.2 μmol/L of paclitaxel, and Western blot analysis was done to determine the protein expression levels of Aurora‐A, FOXM1 and GAPDH. B, Growth curves of control cells and Aurora‐A overexpression cells that were treated with 0.2 μmol/L of paclitaxel. Cells were harvested and counted at indicated time. C, Parental and paclitaxel‐resistant MDA‐MB‐231 cells were treated with 0.2 μmol/L of paclitaxel for 0, 12, 24, 48 and 72 h. Protein lysates were analysed by Western blotting using specific antibodies. D, Parental and paclitaxel‐resistant MDA‐MB‐231 cells were treated with 0.2 μmol/L of paclitaxel. Cells were counted at indicated time to evaluate cell viability. E, Paclitaxel‐resistant MDA‐MB‐231 cells were treated with different drug alone or in combination. Western blot was performed to analyse the Aurora‐A and FOXM1 level. GAPDH used as loading control. F, Paclitaxel‐resistant MDA‐MB‐231 cells were treated with different drug alone or combination. MTT assay was performed to evaluate cell viability. Bars, ±SD. ***P < .001

Previous study reported that FOXM1 mediates resistance to paclitaxel.^22^ We sought to investigate whether increased Aurora‐A induces resistance to paclitaxel. To prove the hypothesis, we stably introduced Aurora‐A expression cDNA in MDA‐MB‐231 cells. Cells were treated with 10 μg/mL of paclitaxel for 0, 24, 48, 72 or 96 hours. Western blot of Aurora‐A and FOXM1 levels showed that in control cells the levels of both Aurora‐A and FOXM1 decreased with treatment at 72 hours, whereas there is no significant decrease of both Aurora‐A and FOXM1 in Aurora‐A overexpression cells (Figure 6A, lane 4, 5 vs lane 9, 10). Growth curves showed that the overexpression of Aurora‐A led to significant increase in cell viability upon paclitaxel treatment (Figure 6B). To further investigate the role of Aurora‐A in paclitaxel resistance, we generated paclitaxel‐resistant cell line. Aurora‐A levels in parental and resistant lines were analysed by Western blot. Interestingly, both Aurora‐A and FOXM1 levels were elevated in paclitaxel‐resistant cell lines. After treatment with paclitaxel, Aurora‐A and FOXM1 levels simultaneously reduced in parental cells at 48 and 72 hours, whereas in paclitaxel‐resistant cells there is no significant change (Figure 6C, lane 2, 3, 4 vs lane 6, 7, 8). Growth curves also revealed significant differences between parental and resistant cells (Figure 6D). These data indicate that Aurora‐A can protect breast cancer cells from treatment with paclitaxel.

Studies from ours and others reported that the antibiotic drug thiostrepton targeted FOXM1 and greatly inhibited growth of TNBC cells.^23^, ^24^ We sought to explore whether thiostrepton could reverse paclitaxel resistance. Notably, both Aurora‐A and FOXM1 were reduced when treated with thiostrepton alone or in combination with paclitaxel (Figure 6E, lane 4 and lane 5). However, combination treatment with paclitaxel and Aurora‐A kinase inhibitor VX‐680 has slight effect on Aurora‐A and FOXM1 levels. Proliferative assays showed that paclitaxel‐resistant cells exhibited a significant decrease in cell viability when treated with thiostrepton alone or in combination with paclitaxel (Figure 6F). These results indicated that Aurora‐A confers resistance by up‐regulating FOXM1. It is worth to mention that Aurora‐A kinase inhibitor VX680 in combination with paclitaxel can significantly inhibit growth of resistant cells, which indicated other mechanisms may account for VX680 effect.

## 4 DISCUSSION

We reported here that Aurora‐A promotes proliferation of TNBC cells by stabilizing FOXM1. Knock‐down of Aurora‐A significantly suppressed cell proliferation and colony formation in TNBC cell lines. Notably, Aurora‐A‐mediated reduced colony formation was rescued by FOXM1 overexpression, indicating that the reduction in FOXM1 levels is the critical mechanism by which depletion of Aurora‐A inhibits proliferation, and this function is critical for the growth of TNBC cells. We also observed that aberrant expression of FOXM1 and Aurora‐A is highly correlated in clinical TNBC samples, providing clinical evidence for Aurora‐A regulating FOXM1 in TNBC. Depletion of Aurora‐A in MDA‐MB‐231 cells not only reduced the steady‐state levels of FOXM1 protein but also led to decrease in FOXM1 mRNA levels (Figure 4D), arguing that Aurora‐A regulates FOXM1 level via both transcriptional and posttranscriptional mechanisms. Interestingly, in TNBC cells the kinase activity of Aurora‐A is not involved in this function of Aurora‐A, whereas in ER‐positive cells, such as MCF‐7, the kinase activity of Aurora‐A is probably important, as FOXM1 is obviously down‐regulating by Aurora‐A kinase inhibitor VX680. Thus, inhibition of Aurora‐A kinase activity using small molecules may not enough to block a critical oncogenic function of Aurora‐A in triple‐negative breast cancer. Indeed, several studies were currently being evaluated in preclinical models of TNBC.^25^, ^26^, ^27^, ^28^ Some models are sensitivity to ENMD‐2076, a kinase inhibitor of Aurora‐A, by induction of apoptosis, whereas some models exhibiting intrinsic or acquired resistance to treatment.^26^ The result from our study suggested that inhibition of both kinase‐dependent and kinase‐independent function is required for TNBC patients.

Several mechanisms are considered responsible for the elevated FOXM1 in cancer, including amplification of the FOXM1 locus, increased stability of FOXM1 and enhanced transcription of FOXM1.^29^ FOXM1 stability or expression in cancer cells can be increased via the interaction with different types of proteins. For example, the Wnt signalling pathway inhibits FOXM1 degradation,^30^ and the direct interaction with nucleophosmin and phosphorylation by Chk2 complexes stabilizes FOXM1 protein.^31^, ^32^ E2F, c‐Myc and hypoxia‐inducible factor‐1 directly bind to the FOXM1 promoter and stimulate its expression.^33^, ^34^, ^35^ Previously, we reported that kinase‐dead Aurora‐A could effectively transactivate the FOXM1 promoter through a Forkhead response element and promote the self‐renewal of breast cancer stem cell.^17^ In the present study, we demonstrated that in TNBC cells, Aur‐A binds to and stabilizes FOXM1 by attenuating its ubiquitin. FOXM1 is a key regulator of cell cycle.^36^ Up‐regulation of FOXM1 in cancer cells leads to uncontrolled cell division and genomic instability.^29^, ^37^, ^38^ In normal cell cycle, FOXM1 is synthesized and degraded in every cycle of cell division. It is reported that FOXM1 is polyubiquitinated by APC/CCdh1 for degradation by the proteasome in late mitosis and early G1‐phase.^20^ The degradation of FOXM1 in the late mitosis and early G1‐phase is important for regulated entry into S phase. Previous study reported that FOXM1 exhibited obvious reduction after 3 hours of nocodazole removal (late mitosis and early G1 phase).^20^, ^39^ We observed that the FOXM1 did not exhibit a reduction after 3 hours of nocodazole removal in TNBC cells. However, in Aurora‐A siRNA‐transfected cells, the FOXM1 protein level decreased significantly in late mitosis and early G1 phase of the cell cycle (Figure 1F), suggesting Aurora‐A prevented the loss of FOXM1 in the late mitosis and early G1 phase in TNBC cells. Therefore, the effect of Aurora‐A regulating FOXM1 in TNBC cells is not restricted to the G2/M phase when the kinase activity of Aurora‐A is highest. We concluded that up‐regulation of Aurora‐A led to increased FOXM1 level in cell cycle, with the effect being strongest in the late mitosis and early G1 phase, providing an explanation that Aurora‐A enhanced proliferation of TNBC cells by promoting G1/S transition during cell cycle.

High expression of Aurora‐A was reported a predictive maker for poor prognosis and drug resistance in triple‐negative breast cancer, which is associated with a greater risk of recurrence and relapse.^13^, ^14^, ^40^ To date, chemotherapy remains the only possible therapeutic option in the treatment of TNBC.^2^, ^4^, ^41^, ^42^, ^43^, ^44^, ^45^ Paclitaxel is a commonly used chemotherapeutic agent for TNBC. However, some of the patients undergone recurrence after short‐term respond and many others fail to respond to this drug, indicating that TNBC cells can possess either acquired or inherent resistance to this drug which poses a significant clinical challenge. Previous study from our and other laboratory reported that Aurora‐A plays an important role in drug resistance.^46^, ^47^, ^48^ Aurora‐A stabilizes survivin in gastric cancer to promote drug resistance.^49^ High expression level of Aurora‐A was involved in tamoxifen resistance.^46^ In the present study, we demonstrated that high levels of Aurora‐A offer TNBC cells an additional growth advantage and protection against paclitaxel. Consistently, the paclitaxel‐resistant TNBC cells exhibit high expression of Aurora‐A and cannot be down‐regulated by paclitaxel compared to parental cells. The mechanisms by which cells acquire resistance are multiple and complex. It has been shown that FOXM1 mediated resistance to chemotherapeutic drugs in breast cancer cells.^22^, ^50^ Jimmy et al reported that cisplatin‐resistant breast cancer cells could be reversed by the FOXM1 inhibitor thiostrepton.^22^ We observed that FOXM1 was also up‐regulated in paclitaxel‐resistant cells. Combination of FOXM1 inhibitor thiostrepton and paclitaxel treatment can reverse acquired paclitaxel resistance in TNBC cells and significantly inhibiting cell proliferation. These results show the likely mechanism by which Aur‐A confers resistance is by preventing the degradation of FOXM1, and this function is kinase independent. A study using in vivo model of triple‐negative breast cancer reported that Aurora‐A kinase inhibitor MLN8237 and taxanes have synergistic antitumour activity.^51^ We also found a synergistic activity between VX680 and paclitaxel in TNBC cells, indicating other mechanisms are involved in the antitumour activity of aurora kinase inhibitor. Interestingly, the Aurora‐A and FOXM1 could be simultaneously reduced by thiostrepton in paclitaxel‐resistant TNBC cells. Together, our result suggested that thiostrepton inhibited growth of paclitaxel‐resistant cells by down‐regulating both Aurora‐A and FOXM1 and could be a therapeutic strategy for reversing paclitaxel chemoresistance in TNBC patients.

Overall, the mechanism we revealed in this study provided insight into an additional pathway through which Aurora‐A regulates FOXM1 in triple‐negative breast cancer. Aurora‐A‐mediated stabilization of FOXM1 could reflect its kinase‐independent role in enhanced proliferation capacity of TNBC cancer cells. Although inhibition of Aurora‐A kinase was a promising regimen for TNBC cancer therapy, the results presented here suggested that the use of an antibiotic drug thiostrepton, which is capable of directly down‐regulating Aurora‐A and FOXM1 level, could synergize with paclitaxel to reverse paclitaxel chemoresistance in TNBC. Our findings provide evidence for using thiostrepton as a new therapeutic strategy for paclitaxel‐resistant TNBC patients.

## CONFLICT OF INTEREST

All authors of this research paper have directly participated in the planning, execution or analysis of the study. All authors of this paper have read and approved the final version submitted. The contents of this manuscript have not been copyrighted or published previously. The contents of this manuscript are not now under consideration for publication elsewhere. The contents of this manuscript will not be copyrighted, submitted or published elsewhere while acceptance by the Journal is under consideration. There are no directly related manuscripts or abstracts, published or unpublished, by any author(s) of this paper.

## AUTHOR CONTRIBUTION

NY, QL and BLX designed the study. NY and CW performed the experiments. JW and DH provided the clinical sample. ZFW, MY and MK analysed the data. NY, QL and BLX wrote the paper.

## Data Availability

All data used for this project are publicly available and accessible online.
